# Intraclass correlation coefficients in the Brazilian network for surveillance of severe maternal morbidity study

**DOI:** 10.1186/1471-2393-12-101

**Published:** 2012-09-21

**Authors:** Samira M Haddad, Maria H Sousa, Jose G Cecatti, Mary A Parpinelli, Maria L Costa, Joao P Souza

**Affiliations:** 1 Department of Obstetrics & Gynecology, University of Campinas, R Alexander Fleming, 101, Campinas, SP, 13083-881, Brazil; 2 Campinas Center for Studies in Reproductive Health (CEMICAMP), Campinas, SP, Brazil; 3 Department of Reproductive Health and Research, World Health Organization, UNDP/UNFPA/WHO/World Bank Special Programme of Research, Development and Research Training in Human Reproduction, Geneva, Switzerland

**Keywords:** Intraclass correlation coefficient, Maternal near miss, Severe maternal morbidity, Maternal and perinatal health

## Abstract

**Background:**

The purpose of the study was to evaluate intraclass correlation coefficients (ICC) of variables concerning personal characteristics, structure, outcome and process in the Brazilian Network for Surveillance of Severe Maternal Morbidity study conducted to identify severe maternal morbidity/near miss cases using the World Health Organization criteria.

**Method:**

It was a cross-sectional, multicenter study involving 27 hospitals providing care for pregnant women in Brazil. Cluster size and the mean size of the primary sampling unit were described. Estimated prevalence rates, ICC, their respective 95% confidence intervals, the design effect and the mean cluster size were presented for each variable.

**Results:**

Overall, 9,555 cases of severe maternal morbidity (woman admitted with potentially life-threatening conditions, near miss events or death) were included in the study. ICC ranged from < 0.001 to 0.508, with a median of 0.035. ICC was < 0.1 for approximately 75% of the variables. For process-related variables, median ICC was 0.09, with 0.021 for those related to outcome. These findings confirm data from previous studies. Homogeneity may be considered minor, thus increasing reliability of these findings.

**Conclusions:**

These results may be used to design new cluster trials in maternal and perinatal health and to help calculate sample sizes.

## Background

Cluster studies are widely used in epidemiological research to evaluate health interventions and implement public policies. In these cases, selection units or randomization units consist of population groups (specific geographical areas) or healthcare units (hospitals) or healthcare sectors rather than individuals [1,2].

In single-stage cluster sampling, all subjects belonging to each group are included to obtain data of interest. Unlike simple random sampling (SRS) in which each individual has an equal likelihood of being selected within the general population, data obtained from clusters may not be sufficiently representative to allow for generalization. This is due to a greater degree of homogeneity in characteristics of the population under observation, as opposed to heterogeneity found in the general population [2].

Reliability of estimates obtained from studies using cluster sampling may be analyzed by measuring inter- and intra-cluster variance. One way to perform these measurements is to calculate δ, the intraclass correlation coefficient (ICC), for variables evaluated in the study [3]. Another way to perform measurements is to calculate the design effect (DEFF).

ICC is a coefficient that measures the degree of homogeneity among elements within clusters. The ICC of a variable indicates to what extent variance of a parameter can be explained by variation between clusters [4,5]. Its value depends on the type of variable, cluster size and prevalence of the condition [6]. Coefficients closest to zero suggest intraclass heterogeneity, indicating that the variable is randomly distributed among clusters. Similarly, values close to 1 indicate homogeneity in a sample and the variance of cluster units is greater than that of single elements [2].

Design effect (DEFF) indicates the extent to which the variance of parameter estimate is a result of study design, in this case the cluster sampling, compared to what would be obtained if sampling had been carried out by the SRS method [7]. For example, a DEFF value of 3 indicates that the variance of parameter estimation is three times greater than would be found if the study had been based on a random sample of equal size. Components involved in calculating the DEFF are ICC and mean cluster size. The larger the ICC and the larger the cluster, the higher the DEFF value [8].

The routine adoption of ICC calculation in cluster studies may improve interpretation of the results and facilitate the development of new studies in the field. These values could be used as a correction factor for the calculation of sample size in future cluster studies, thus avoiding underestimates, since, in studies in which SRS is used, the sample size required to achieve sufficient statistical power is generally smaller [4].

The Brazilian Network for Surveillance of Severe Maternal Morbidity study was developed with the objective of identifying cases of severe maternal morbidity/near miss (woman admitted with potentially life threatening conditions, near miss) or death in 27 hospitals distributed throughout Brazil. The participating centers were selected using the following criteria: the availability of each center to participate in the surveillance study, the geographical region of the country in which the hospital was situated and a requirement that the hospital in question performed at least 1,000 deliveries per year. Therefore, the participating centers constituted the primary sampling units, while the subjects at each center represented the units of analysis [9].

The objective of the present report is to evaluate the intraclass correlation coefficients of the variables associated with outcome, process, personal characteristics and structure, based on the data collected by the Brazilian Network for Surveillance of Severe Maternal Morbidity, as well as the design effects.

## Methods

The Brazilian Network for Surveillance of Severe Maternal Morbidity, established in 2009, conducted a cross-sectional, multicenter study involving 27 hospitals providing care for pregnant women in Brazil. The objective of this network was to identify cases of severe maternal morbidity/near miss, using the criteria established by the World Health Organization (WHO) to characterize these conditions [10]. According to this definition, a maternal near miss is a woman who experienced a very serious complication during pregnancy and almost died but survived at least until the 42^nd^ day of postpartum period [10].

The selection of clusters was based predominantly on the location of the hospitals in order to ensure broad coverage throughout the country, and on a minimum of 1,000 deliveries per center per year to enable the calculated sample size to be reached. The sample size calculation was based on an prevalence of 8 cases of near miss for every 1,000 deliveries [11], a maternal death ratio of 140 for every 100,000 liveborn infants, with confidence level of 95%, and considering a precision level of 8/1,000 for near miss and 8.5/1,000 for maternal death; it was added approximately 25% based on the fact that this definition of near miss had not yet been tested. In result, around 75,000 deliveries would have to be monitored to identify approximately 600 cases of near miss and 100 maternal deaths. The study was evaluated and approved locally by the respective Institutional Review Board of each participating hospital and also by the National Council for Ethics in Research.

Over the course of one year, all the pregnant women admitted to these institutions were monitored. Women found to have any of the morbidity criteria were included in the analysis. The data of interest to the study were collected from the women’s charts immediately after they were discharged from hospital [12]. After data collection was complete in June 2010, procedures were initiated to verify and correct any inconsistencies to ensure the quality of the data obtained. These procedures included daily check of all data inputted in the database, appointment of possible inconsistence to researchers, correction of errors and programmed check of all dataset at the end of study.

### Data analysis

Initially, the cluster size and the mean size of the primary sampling unit (PSU) obtained from the total sample of 9,555 women were described. Estimated prevalences of each dichotomized variable, intraclass correlation coefficients (ICC), their respective 95% confidence intervals (95% CI), design effects (DEFF) and mean cluster size for each variable were calculated. The software programs used for the analysis were SPSS, version 17.0 [13] and Stata, version 7.0 [14], taking into consideration the cluster sampling plan (centers) for data analysis. Information on health facilities and live births for the whole country came from official data [15,16].

### Sampling plan used in the Brazilian network for surveillance of severe maternal morbidity study

A single-stage cluster sampling was used, with 27 primary sampling units (PSU) corresponding to the 27 participating centers (hospitals). The sampling plan did not involve stratification of the PSU or weighting of the data. The unit of analysis (subject) was the registration of each woman admitted with potentially life threatening conditions, near miss or death.

#### Prevalence – ratio estimator *(r)*[2]

(1) $$r=\frac{y}{x}=\frac{\sum y_{\alpha }}{\sum x_{\alpha }}\;=\;\frac{\sum_{\alpha =1}^{a}\sum_{\beta =1}^{X_{\alpha }}y_{\alpha \beta }}{\sum_{\alpha =1}^{a}x_{\alpha }}$$

in which $y=\sum_{\alpha }y_{\alpha }$= $\sum_{\alpha }\sum_{\beta }y_{\alpha \beta }$(α for cluster and β for individual) is the total number of subjects in the sample possessing a certain category (for example “Yes”) for the variable *y*_*αβ*_ (dichotomized); for example, *y*: the total number of subjects in the sample with “prenatal care at the same facility”, and $x=\sum_{\alpha }x_{\alpha }$ is the size of the available sample (valid) for that variable, where x_α_ is the sample size for the cluster ‘α’.

For this study, the ratio estimator is:

(2) $$r=\frac{y}{x}=\frac{\sum_{\alpha =1}^{27}y_{\alpha }}{\sum_{\alpha =1}^{27}x_{\alpha }}$$

#### Intraclass correlation coefficient – ICC (Roh)

According to Kish [2], the intraclass correlation coefficient is:

$Roh=\frac{s_{a}^{2}-s_{b}^{2}/b}{{\hat{s}}^{2}}$, where $s_{a}^{2}$ is the variance between clusters; $s_{b}^{2}$ is the variance within clusters; b is the size of clusters and ${\hat{s}}^{2}$is the estimate of $S_{.}^{2}$ (the variance in individual level). The estimate ${\hat{s}}^{2}$is obtained by: ${\hat{s}}^{2}=s_{a}^{2}+\frac{b-1}{b}s_{b}^{2}$

Stata’s equivalent computing formula [14] is:

(3) $$ICC=\frac{\left(F-1\right)a/n}{1+\left(F-1\right)a/n}$$

where ‘F’ is the Snedecor’s F-value from the ANOVA table and ‘a’ is the number of groups. The variance estimate for ICC is obtained by an extensive asymptotic formula, and because this it was not showed.

#### Design effect - DEFF [2]

(4) $$Deff\;=\frac{{\mathrm{var}}_{\mathrm{actual}}\left(r\right)}{{\mathrm{var}}_{\mathrm{SRS}}\left(r\right)}=\frac{s_{a}^{2}/a}{s^{2}/n}$$

where var_actual_(r) is the estimated variance according to the complex design being studied and var_SRS_(r) is the variance in the estimator considering the design as if it were calculated using a SRS of the same size, *n*.

Variance estimator of *r* under the design being studied:

(5) $${\mathrm{var}}_{\mathrm{actual}}\left(r\right)=\frac{\frac{1}{\left(a-1\right)}\sum_{a=1}^{a}{\left(r_{a}-r\right)}^{2}}{a}$$

Variance estimator of *r* under SRS:

(6) $${\mathrm{var}}_{\mathrm{SRS}}\left(r\right)=\frac{r\left(1-r\right)}{\left(n-1\right)}$$

where *r* is the ratio estimator under the SRS.

#### Recalculation of the sample size

Using the ICC from the main outcome as a correction factor [17]:

(7) $$\text{n}*=a/{\text{ICC}}^{.}\left(\text{Deff}-1+\text{ICC}\right)$$

Where ‘a’ is the number of clusters, ‘Deff’ the design effect and ‘ICC’ the intraclass correlation coefficient of the main outcome.

## Results

Of the 82,388 deliveries performed in the participating institutions during the study period, 82,144 liveborn infants and 9,555 cases of maternal morbidity were included in the study. The mean size of each cluster was 354 and the distribution of cases per center and region and of the liveborn infants per region is shown in Table 1.

**Table 1:** Distribution of cases and live births (LB) by cluster and according to geographical region of the country

**Region**	**Center**	**n**_**c **_**(or x**_**α **_**#)**	**LB in the network**	LB in Brazil*
**Southeast n**_**se**_**= 2,794 (29.2%)**	1	48	37,865 (46.1%)	1,130,407 (38.5%)
	2	59		
	3	66		
	4	74		
	5	96		
	6	112		
	7	154		
	8	155		
	9	172		
	10	186		
	11	253		
	12	369		
	13	1050		
**Northeast n**_**ne**_**= 5,148 (53.9%)**	1	118	33,172 (40.4%)	888,268 (30.3%)
	2	210		
	3	263		
	4	281		
	5	294		
	6	465		
	7	566		
	8	920		
	9	945		
	10	1086		
**South n**_**s**_**= 939 (9.8%)**	1	98	4,532 (5.5%)	371,497 (12.7%)
	2	841		
**Midwest n**_**mw**_**= 609 (6.4%)**	1	609	2,527 (3.1%)	222,658 (7.6%)
**North n**_**n**_**= 65 (0.7%)**	1	65	4,048 (4.9%)	321,998 (11.0%)
**Total (n)**	27	9555	82,144	2,934,828

Mean cluster size (n_a_)= 354.

#Denominator of the expression of the ratio estimator.

*Data from SINASC (Brazil, 2011) [16].

The ICCs ranged from < 0.001 to 0.508, with a median of 0.035. The ICC was below 0.1 for approximately 75% of the variables. In this block of variables, the mediann ICC was 0.021, while in the other 25%, median ICC was 0.195. The ICCs for the variables related to the process are shown in Table 2, with values ranging from 0.001 to 0.508 (median 0.09), while DEFF values varied from 1.5 to 292.63 (median 20.52).

**Table 2:** Estimates of prevalence, intraclass correlation coefficients, their respective 95% CI, design effect, and mean cluster size for variables related to the process

**Variable**	**P (%)**	**ICC**	**95% CI for ICC**	**DEFF**	**n**_**a**_
· Prenatal care at the same institution	22.1	0.103	0.033−0.174	44.72	316
· Access of women to the center (Spontaneous demand)	47.9	0.195	0.078−0.312	118.12	333
· Major type of healthcare insurance used for prenatal care (Public)	88.5	0.115	0.039−0.191	30.27	279
· Major type of healthcare insurance used for hospital admission (Public)	98.9	0.508	0.323−0.693	31.66	354
Procedure related to abortion					
· Procedure performed: D&C	77.8	0.054	0.001−0.148	1.50	9
· Procedure performed: Oxytocin	33.3	0.339	0.136−0.542	9.39	9
· Procedure performed: Vacuum aspiration	7.0	0.001	<0.001−0.068	2.26	9
· Procedure performed: Prostaglandins	22.2	0.118	<0.001−0.245	1.91	9
· Procedure performed: Other	7.0	0.155	0.011−0.298	1.53	9
Related to PLTC criteria					
· Any therapeutic management	76.1	0.272	0.125−0.419	225.19	354
· Transfusion of blood derivatives	16.4	0.079	0.024−0.133	37.50	354
· Central venous access	3.8	0.090	0.028−0.151	21.23	354
· Admission to ICU	22.1	0.288	0.136−0.439	199.58	354
· Prolonged hospital stay	30.0	0.098	0.032−0.164	81.88	354
· Intubation unrelated to anesthesia	3.1	0.047	0.013−0.081	12.99	354
· Return to operating room	3.3	0.021	0.005−0.038	11.75	354
· Hysterectomy/laparotomy	6.2	0.043	0.011−0.074	20.44	354
· Use of magnesium sulfate	48.3	0.363	0.192−0.534	292.63	354
· Another major surgical procedure	0.8	0.008	0.001−0.016	6.35	354
Related to near miss criteria					
· Any management near miss criteria	6.1	0.075	0.023–0.127	20.52	354
· Continuous use of vasoactive drug	2.6	0.028	0.007–0.049	8.93	354
· Hysterectomy due to infection or hemorrhage	1.8	0.031	0.008–0.054	8.05	354
· Transfusion of ≥5 U red cells	2.6	0.053	0.015–0.091	11.42	354
· Intubation and ventilation ≥ 60 min unrelated to anesthesia	3.1	0.047	0.013–0.082	13.94	354
· Dialysis for acute renal failure	0.7	0.009	0.001−0.017	3.60	354
· Cardiopulmonary resuscitation (CPR)	1.3	0.018	0.004–0.033	8.41	354
· At least one of these conditions was already present when the woman was admitted	32.0	0.132	0.046–0.218	6.91	34
Related to delay in care					
· Delay related to patients and/or their family members (Yes/No)	39.3	0.090	0.028−0151	27.26	310
· Search for a health service	5.3	0.174	0.067–0.281	54.03	310
· Geographic difficulty in accessing health service	2.4	0.352	0.183–0.520	113.16	310
· Refused treatment/care	5.1	0.014	0.002–0.025	7.72	310
· Prenatal care absent or inadequate	32.1	0.043	0.012–0.075	14.98	310
· Delay in care related with health professional	17.3	0.328	0.164−0.492	134.27	331
· Delay in diagnosis	5.5	0.218	0.091–0.346	72.81	331
· Delay in starting treatment	6.7	0.321	0.159–0.483	105.23	331
· Inadequate management	13.6	0.300	0.144–0.456	121.82	331
· Delay in referring or transferring the case	3.3	0.055	0.015–0.095	20.77	331

P: Prevalence of each category; ICC: Intraclass Correlation Coefficient; 95% CI: 95% confidence interval; DEFF: Design effect; n_a_: Mean cluster size; PLTC: Potentially life-threatening condition; NM: Near miss.

Table 3 shows the variables related to outcome, with ICCs that ranged from < 0.001 to 0.375 (median 0.021) and DEFF values of 0.94 to 289 (median 6.24). ICC values were < 0.05 for approximately 74% of the variables associated with outcome, while the proportion of variables for which ICC was < 0.07 was almost 80%.

**Table 3:** Estimates of prevalence, intraclass correlation coefficients, their respective 95% CI, design effect and mean cluster size for variables related to outcome

**Variable**	**P (%)**	**ICC**	**95% CI for ICC**	**DEFF**	**n**_**a**_
· Any hemorrhagic complication	24.8	0.165	0.062−0.268	129.54	354
· Abruptio placentae	5.7	0.016	0.003−0.029	9.55	354
· Placenta previa/acreta	2.2	0.017	0.004−0.031	6.24	354
· Complicated ectopic pregnancy	3.1	0.034	0.009−0.059	17.72	354
· Uterine rupture	0.3	0.005	<0.001−0.011	2.12	354
· Severe post abortion hemorrhage	1.3	0.015	0.003−0.028	5.92	354
· Postpartum hemorrhage	12.5	0.248	0.110−0.387	200.97	354
✓ Atony	63.0	0.375	0.134−0.617	77.37	44
✓ Retained placenta	11.8	0.114	0.005−0.224	12.94	44
✓ Tears	8.8	0.130	0.008−0.251	10.04	44
✓ Coagulopathy	4.6	0.102	0.002−0.203	13.51	44
✓ Uterine inversion	0.6	0.035	<0.001−0.079	1.65	44
✓ Other obstetric cause	11.2	0.370	0.130−0.610	43.77	44
· Other severe hemorrhage	1.0	0.023	0.005−0.041	6.16	354
· Any complication due to hypertension	70.2	0.183	0.072−0.294	124.72	354
· Severe preeclampsia	51.2	0.231	0.099−0.363	188.23	354
· Eclampsia	4.5	0.023	0.005−0.040	14.62	354
· Severe hypertension	18.3	0.334	0.169−0.499	289.00	354
· HELLP syndrome	6.2	0.028	0.007−0.049	15.43	354
· Acute fatty liver	0.2	0.005	0.001−0.010	1.59	354
· Any other complication	17.2	0.103	0.034−0.172	31.93	354
· Pulmonary edema	1.7	0.020	0.004−0.036	4.45	354
· Seizures	1.4	0.009	0.001−0.016	3.66	354
· Thrombocytopenia	3.9	0.022	0.005−0.039	6.77	354
· Thyrotoxic crises	0.1	0.007	0.001−0.013	1.55	354
· Shock	3.1	0.034	0.009−0.059	9.95	354
· Acute respiratory failure	4.0	0.051	0.014−0.087	19.37	354
· Acidosis	1.6	0.021	0.005−0.038	12.54	354
· Cardiopathy	1.7	0.013	0.002−0.023	5.71	354
· Stroke	0.2	0.001	0.001−0.003	1.10	354
· Coagulation defects	2.0	0.012	0.002−0.021	4.69	354
· Disseminated intravascular coagulation	0.6	0.012	0.002−0.021	2.60	354
· Thromboembolism	0.9	0.010	0.001−0.018	4.95	354
· Diabetic ketoacidosis	0.2	0.004	0.001−0.008	0.94	354
· Jaundice/liver dysfunction	1.4	0.014	0.003−0.026	2.46	354
· Meningitis	<0.1	<0.001	0.001−0.002	0.94	354
· Severe sepsis	3.5	0.064	0.019−0.109	18.09	354
✓ Postpartum endometritis	19.5	0.131	0.011−0.252	3.95	13
✓ Post abortion endometritis	11.0	0.043	0.001−0.115	2.13	13
✓ Pulmonary focus	29.3	0.055	0.001−0.133	2.03	13
✓ Urinary focus	25.0	0.013	0.001−0.067	1.08	13
✓Other	15.2	0.042	0.001−0.113	1.15	13
· Acute renal failure	2.2	0.027	0.006−0.047	8.68	354
· Complication possibly associated with Influenza A ‘H1N1’	2.2	0.056	0.016−0.097	20.62	354
· Any clinical maternal near miss criteria	5.5	0.037	0.010−0.065	15.44	354
· Cyanosis	1.3	0.023	0.005−0.040	12.63	354
· Gasping	0.4	0.009	0.001−0.017	4.21	354
· Respiratory rate <6 or RR>40	2.0	0.016	0.003−0.029	8.42	354
· Shock	2.6	0.031	0.008−0.054	9.24	354
· Oliguria unresponsive to fluids or diuretics	0.9	0.010	0.001−0.018	4.43	354
· Coagulation problems	1.0	0.012	0.002−0.021	4.88	354
· Loss of consciousness for 12h or more	0.7	0.005	<0.001−0.010	4.25	354
· Absence of consciousness and absence of pulse rate/heartbeat	0.7	0.006	0.001−0.011	4.96	354
· Stroke	0.3	0.002	0.001−0.005	1.16	354
· Uncontrolled seizures – total paralysis	0.2	0.001	<0.001−0.004	2.22	354
· Jaundice in presence of preeclampsia	0.3	0.001	<0.001−0.002	1.36	354
· Any laboratory near miss criteria	5.2	0.047	0.013−0.082	12.08	354
· O_2_ saturation < 90% for longer than 60 min	1.9	0.016	0.003−0.029	8.30	354
· PaO_2_/FiO_2_ < 200 (Yes/No)	1.1	0.015	0.003−0.028	8.37	354
· Creatinine ≥ 300 mmol/l or ≥ 3.5 mg/dl	1.0	0.007	0.001−0.013	3.03	354
· Bilirubin ≥ 100 mmol/l or ≥ 6 mg/dl	0.5	<0.001	<0.001−0.002	1.25	354
· pH < 7.1	0.8	0.009	0.001−0.016	5.32	354
· Lactate > 5	0.8	0.098	0.032−0.165	18.31	354
· Platelet < 50,000	2.1	0.025	0.006−0.044	5.32	354
· Absence of consciousness plus glucose and ketoacids in urine	0.2	0.004	<0.001−0.009	3.49	354
· Condition of woman at discharge (Medical discharge)	94.1	0.011	0.002–0.020	6.54	354
· Unsafe abortion	0.6	0.025	0.006–0.045	7.43	310
· **Maternal Death**	1.5	0.018	0.004−0.033	7.93	354
· **Maternal Death + NM**	9.5	0.077	0.023−0.130	21.09	354

P: Prevalence of each category; ICC: Intraclass Correlation Coefficient; 95% CI: 95% confidence interval; DEFF: Design effect; n_a_: Mean cluster size; NM: Near miss.

Tables 4 and 5 show the ICC values with their respective 95% confidence intervals, prevalence rates, DEFF values and the mean size of the clusters for the variables referring to population/obstetric characteristics and structure, respectively. With the exception of some variables related to structure, particularly those associated with delays in the service or healthcare system, ICC values were very low.

**Table 4:** Estimates of prevalence, intraclass correlation coefficients, their respective 95% CI, design effect and the mean cluster size with respect to personal and obstetric data

**Variable**	**P (%)**	**ICC**	**95% CI for ICC**	**DEFF**	**n**_**a**_
· Age (<30 years)	65.6	0.013	0.002−0.024	4.00	354
· Skin color (White)	42.5	0.285	0.127−0.443	146.55	264
· Schooling (Primary)	46.5	0.051	0.012−0.091	17.45	256
· Marital status (Has a partner)	53.2	0.176	0.066−0.286	101.96	298
· Weight (<75 kg)	52.3	0.055	0.010−0.101	21.45	167
· Height (<1.60 m)	48.6	0.054	0.007−0.101	13.51	151
· Body Mass Index (Low BMI)	15.4	0.053	0.006−0.100	18.47	146
· Number of pregnancies (One)	41.9	0.010	0.001−0.019	9.01	352
· Number of deliveries (None)	48.2	0.010	0.002−0.019	8.87	352
· Number of abortions (None)	77.6	0.005	<0.001−0.011	2.59	352
· Number of previous C-sections (None)	76.0	0.011	0.002−0.021	6.87	347
· Number of live births (None)	51.3	0.016	0.003−0.028	11.27	341
· Time since last delivery (≤2 years)	25.5	0.022	0.001−0.043	4.23	99
· Previous uterine surgery	2.0	0.067	0.017−0.117	20.52	299
· Number of prenatal visits (<6)	46.4	0.018	0.003−0.034	5.34	276
· Pregnant at admission	95.0	0.043	0.011−0.074	20.70	354
· Gestational age at admission (<37 weeks)	53.4	0.069	0.020−0.117	37.71	327
· Mode of onset of labor (no labor)	49.4	0.113	0.038−0.188	88.44	349
· Gestational age at resolution (<37 weeks)	45.7	0.055	0.015−0.095	31.28	309
· Mode of delivery (C-section before labor)	49.6	0.111	0.037−0.184	87.28	352
· Mode of onset of abortion (Spontaneous)	63.6	0.044	<0.001−0.141	1.52	8
· Total number of fetuses (None)	6.0	0.128	0.044−0.212	52.87	325
· Fetal presentation at birth (Cephalic)	91.3	0.011	0.001−0.020	4.77	273
· Some pathological or risky condition prior to this current pregnancy	48.9	0.115	0.038−0.191	47.12	305
· Chronic hypertension	17.7	0.046	0.012−0.079	15.98	305
· Obesity	24.1	0.110	0.036−0.184	65.40	305
· Low weight	0.3	0.004	<0.001−0.008	1.68	305
· Diabetes mellitus	2.5	0.023	0.005−0.041	7.84	305
· Smoking	5.7	0.065	0.018−0.111	44.05	305
· Cardiac diseases	2.9	0.025	0.006−0.045	9.23	305
· Respiratory diseases	2.8	0.018	0.004−0.033	9.67	305
· Renal diseases	1.2	0.038	0.010−0.067	11.74	305
· Sickle cell disease-thalassemia	0.8	0.005	<0.001−0.011	5.08	305
· HIV/AIDS	1.1	0.006	<0.001−0.013	3.16	305
· Thyroid disease	1.4	0.008	0.001−0.016	2.65	305
· Neurologic diseases/epilepsy	1.2	0.011	0.001−0.020	4.35	305
· Collagenosis	0.6	0.028	0.007−0.050	8.93	305
· Cancer	0.3	0.006	<0.001−0.012	2.31	305
· Other	5.2	0.037	0.009−0.065	13.99	305
· Drug addiction	1.2	0.005	<0.001−0.010	4.07	305

P: prevalence of each category; ICC: Intraclass Correlation Coefficient; 95% CI: 95% confidence interval; DEFF: Design effect; n_a_: Mean cluster size.

**Table 5:** Estimates of prevalence, intraclass correlation coefficients, their respective 95% CI, design effect and mean cluster size with respect to the variables related to structure

**Variable**	**P (%)**	**ICC**	**95% CI for ICC**	**DEFF**	**n**_**a**_
· Delay related to service or healthcare system	15.6	0.247	0.108−0.387	106.64	328
· Lack of medication	1.3	0.021	0.005–0.038	9.77	328
· Difficulties with municipal/hospital transport	1.3	0.031	0.007–0.055	9.27	328
· Difficulties with communication (hospital/central regulation)	8.8	0.206	0.083–0.329	99.07	328
· Lack of blood derivatives	0.6	0.014	0.002–0.025	8.17	328
· Difficulty related to monitoring (intensive care unit)	4.6	0.230	0.097–0.363	64.99	328
· Lack of trained personnel	3.1	0.063	0.018–0.107	24.25	328
· Difficulty related to access to prenatal care	1.4	0.084	0.025–0.142	20.09	328

P: Prevalence of each category; ICC: Intraclass Correlation Coefficient; 95% CI: 95% confidence interval; DEFF: Design effect; n_a_: Mean cluster size.

Based on the fact that the sample size calculation did not take into account the ICC for the primary outcome of interest, because it was not available from other studies at the time of project development, the sample size was recalculated using the ICC for the variable near miss/deaths as the correction factor.

This was an exercise to evaluate if our sample would have been sufficient for analysis considering the cluster design. This variable was used because it is the main outcome of interest in this study and its value was 0,077. Using the formula for recalculating the sample size in such situations [17], results indicate that a sample size of 7,072 would be needed to identify the near miss/death cases and it corresponds to 74% of the total number of subjects included in the Network.

## Discussion

The values of the intraclass correlation coefficients found in this study can be considered low, close to zero, for the majority of the variables. Intraclass heterogeneity was greater in the variables related to outcome in comparison with the others.

In selecting the clusters, stratification by region was not performed. Proportionally more of the centers were situated in the southeast of the country (48%), and consequently a greater number of liveborn infants were also born in this region (46%). This distribution is in conformity with the actual distribution of healthcare institutions and the proportionality of liveborn infants per region of the country. According to the Ministry of Health’s National Register of Healthcare Institutions, approximately 45% of the institutions registered are situated in the southeastern region [15] and, in 2008, 38.5% of all liveborn infants were born in this region [16]. Proportionality was also maintained in comparison with the other regions.

Although the surveillance of cases of severe maternal morbidity/near miss was prospective, the data were collected from the patients’ charts immediately following the women’s discharge from hospital. Therefore, for some variables, the number of individuals for whom data is available is less than the total number of cases, since it was impossible to recover some of the missing data. This possible loss of part of the data for some of the variables was predicted and taken into consideration in planning the study. Severely ill women may not be able to consent to participate in studies of this type because they may be unconscious, may die or may find themselves in various different situations of emotional fragility. Therefore, data collection following hospital discharge was completely anonymous and avoided the need to obtain informed consent, what allowed a larger number of research subjects to be included, factors that are important in studies of serious conditions with a relatively low prevalence rate.

Previous studies have shown that ICC values are generally higher for variables related to process compared to those for variables related to outcome, since for the same intervention (measure of process), responses may differ between the different individuals under therapeutic management (outcome measurement). Furthermore, higher healthcare levels tend to increase the degree of homogeneity [17-20]. The explanation for this is that when the level of care is higher, there is a greater likelihood that management techniques will be standardized and institutional protocols will be used. In these cases, when one of the objectives is to evaluate compliance with established guidelines, homogeneity may be interpreted as a leveling of institutions with respect to certain recommendations.

This tendency is also seen in the present results, which were obtained from secondary and tertiary care hospitals, the majority of which were teaching hospitals. In this type of institution, the majority of procedures are performed in conformity with evidence-based healthcare protocols. Indeed, the mean ICC value for the variables related to process was 2.6 times higher than the mean ICC value for the variables related to outcome.

The variable with the highest ICC was “Major type of healthcare insurance used for hospital admission” with a value of 0.508. This homogeneity was expected, since only 3 of the 27 centers accepted private patients, all the other centers being exclusively public healthcare services.

Some of the variables related to process were obtained from a specific section of the study focusing on delays during patient care. In addition to obtaining data from the women’s charts, this study also involved another step in which the investigators were instructed to make a subjective analysis of the chain of healthcare services provided, based on the data available on the charts. In addition, after all the variables had been completed, the data for each subject were reanalyzed by the principal investigators and standard procedures for the classification of delays were implemented for all the cases in all the clusters. Therefore, the greater homogeneity found for these variables may be explained by this “standard correction” adopted for all the centers.

Previous studies in the primary care sector have reported ICC values < 0.05 for variables related to outcome and ICC values > 0.05 for variables related to the process [20]. In the field of maternal and perinatal healthcare, Taljaard *et al.* calculated ICC values based on data obtained from secondary/tertiary services [18]. The ICC of some variables analyzed in this study can be compared with ours as reported in Table 6.

**Table 6:** **Comparisom of Intraclass Correlation Coefficients of some variables between this study and study by Taljaard* et al.***[18]

**Variable**	**Haddad*et al.***		**Taljaard*et al.***	
	**ICC**	**n**_**a**_	**ICC**	**n**_**a**_
· HIV positive	0.006	305	0.022	865
· Chronic respiratory conditions	0.018	305	0.042	861
· Diabetes mellitus	0.023	305	0.010	862
· Hysterectomy	0.031	354	0.002	862
· Eclampsia	0.023	354	0.007	862
· Maternal Death	0.018	354	0.0003	866

ICC: Intraclass Correlation Coefficient; n_a_: Mean cluster size.

The findings of those investigators showed that, in general, ICC values for the variables related to the process tended to be > 0.07, with values < 0.07 for the variables related to outcome. The present findings are in agreement with this observation. Furthermore, these values can probably be considered as a good parameter of variance for calculating sample size in new studies in this area, similar to the 0.08 value used by van de Ven *et al.*[21].

Pagel *et al.*[22] estimated ICC of data from five community-based cluster-randomized controlled trials, all evaluating community interventions to improve maternal and newborn health outcomes. The mean cluster size of these studies ranged from 3,934 to 27,953 people. Of 9 key perinatal indicators, only maternal death has the possibility of comparison with our calculations and its ICC ranged from 0.00 to 0.00051.

All those comparisons show what was already known. The smaller the cluster size, the higher the ICC, and the opposite occurs regarding the prevalence of the condition. Mortality events are rare in community based population and the prevalence rises with morbidity association. Pagel *et al.*[22] established as a limitation of their findings that the ICC estimates for rare outcomes as maternal mortality are not likely to be reliable.

Taljaard *et al.*[18] collected data from hospital based population, as our study, and obtained ICC similar to our findings.

This study is based on pregnant women who had at least one of the potentially life threatening conditions. So, prevalence of any factor that is associated with these conditions will be higher in the study sample than in the general obstetric population. This could potentially inflate ICC estimation as ICC generally tends to increase with higher prevalence. The ICCs of this study also depend on other factors such as the hospitals’ characteristics. Caution in applying these ICCs to other settings should be used and this could be considered as a possible limitation of the study.

The absence of previous studies using these new near miss criteria standardized by the WHO [10] made it impossible to obtain the respective ICC values for the variables of interest related to outcome. Therefore, in this study sample size was calculated based exclusively on previous prevalence rates of the condition.

Considering the mean general characteristics of women in this study, most of whom received care in public hospitals linked to universities, and that the ICC values showed sample heterogeneity, studies conducted in middle income countries with similar characteristics to Brazil probably can use the ICC values of this study as the basis for their calculations.

## Conclusions

The Brazilian Network for Surveillance of Severe Maternal Morbidity conducted a pioneering, cross sectional, multicenter study on the application of the new WHO near miss criteria to identify severe cases in obstetrics. This paper reports the intraclass correlation coefficients for study variables. The results found are in agreement with those of previous studies and homogeneity of the data obtained from variables related to outcome may be considered minor (median ICC 0.021), increasing reliability of the study estimates. These values may be used to plan new cluster studies in maternal and perinatal health, mainly studies associated with severe maternal morbidity/near miss events. They may be useful for sample size calculation.

## Abbreviations

ANOVA: Analysis of variance; DEFF: Design effect; ICC: Intraclass correlation coefficient; PSU: Primary sampling unit; SRS: Simple random sampling; WHO: World Health Organization.

## Competing interests

The authors declare that there are no competing interests.

## Authors’ contributions

JPD and JGC were responsible for the original idea of the study. SMH, JGC and MAP were responsible for its implementation. All of them plus the whole group were responsible for care of women, data collection, data consistency and cleaning. MHS, SMH and JGC were responsible for planning and performing analysis. SMH and MHS wrote the first draft of the manuscript which was finalized with output of all others who read and approved the final version.

## Brazilian Network for Surveillance of Severe Maternal Morbidity Group

Fernanda G. Surita, João L. Pinto e Silva, Rodolfo C. Pacagnella, Rodrigo S. Camargo, Vilma Zotareli, Carla Silveira, Lúcio T. Gurgel, Lale Say, Robert C. Pattinson, Marilza V Rudge, Iracema M Calderon, Maria V Bahamondes, Danielly S Santana, Simone P Gonçalves, Eliana M Amaral, Olímpio B Moraes Filho, Simone A Carvalho, Francisco E Feitosa, George N Chaves, Ione R Brum, Gloria C Saint’ynes, Carlos A Menezes, Patricia N Santos, Everardo M Guanabara, Elson J Almeida Jr, Joaquim L Moreira, Maria R Sousa, Frederico A Peret, Liv B Paula, Luiza E Schmaltz, Cleire Pessoni, Leila Katz, Adriana Bione, Antonio C Barbosa Lima, Edilberto A Rocha Filho, Melania M Amorim, Debora Leite, Ivelyne Radaci, Marilia G Martins, Frederico Barroso, Fernando C Oliveira Jr, Denis J Nascimento, Cláudio S Paiva, Moises D Lima, Djacyr M Freire, Roger D Rohloff, Simone M Rodrigues, Sergio M Costa, Lucia C Pfitscher, Adriana G Luz, Daniela Guimaraes, Gustavo Lobato, Marcos Nakamura-Pereira, Eduardo Cordioli, Alessandra Peterossi, Cynthia D Perez, Jose C Peraçoli, Roberto A Costa, Nelson L Maia Filho, Jacinta P Matias, Silvana M Quintana, Elaine C Moises, Fátima A Lotufo, Luiz E Carvalho, Elvira A Zanette, Carla B Andreucci, Márcia M Aquino, Maria H Ohnuma, Rosiane Mattar and Felipe F Campanharo.

## Pre-publication history

The pre-publication history for this paper can be accessed here:

http://www.biomedcentral.com/1471-2393/12/101/prepub
